# m6A Regulator-Mediated Methylation Modification Patterns and Characteristics in COVID-19 Patients

**DOI:** 10.3389/fpubh.2022.914193

**Published:** 2022-05-17

**Authors:** Xin Qing, Qian Chen, Ke Wang

**Affiliations:** ^1^School of Medicine, Southeast University, Nanjing, China; ^2^Clinical Laboratory, Boai Hospital of Zhongshan Affiliated to Southern Medical University, Zhongshan, China; ^3^Department of Pediatrics, The Affiliated Hospital of Southwest Medical University, Luzhou, China

**Keywords:** COVID-19, m6A methylation modification, m6A regulators, diagnostic biomarkers, consensus clustering

## Abstract

**Background:**

RNA N6-methyladenosine (m6A) regulators may be necessary for diverse viral infectious diseases, and serve pivotal roles in various physiological functions. However, the potential roles of m6A regulators in coronavirus disease 2019 (COVID-19) remain unclear.

**Methods:**

The gene expression profile of patients with or without COVID-19 was acquired from Gene Expression Omnibus (GEO) database, and bioinformatics analysis of differentially expressed genes was conducted. Random forest modal and nomogram were established to predict the occurrence of COVID-19. Afterward, the consensus clustering method was utilized to establish two different m6A subtypes, and associations between subtypes and immunity were explored.

**Results:**

Based on the transcriptional data from GSE157103, we observed that the m6A modification level was markedly enriched in the COVID-19 patients than those in the non-COVID-19 patients. And 18 essential m6A regulators were identified with differential analysis between patients with or without COVID-19. The random forest model was utilized to determine 8 optimal m6A regulators for predicting the emergence of COVID-19. We then established a nomogram based on these regulators, and its predictive reliability was validated by decision curve analysis. The consensus clustering algorithm was conducted to categorize COVID-19 patients into two m6A subtypes from the identified m6A regulators. The patients in cluster A were correlated with activated T-cell functions and may have a superior prognosis.

**Conclusions:**

Collectively, m6A regulators may be involved in the prevalence of COVID-19 patients. Our exploration of m6A subtypes may benefit the development of subsequent treatment modalities for COVID-19.

## Introduction

Coronavirus disease 2019 (COVID-19) derived from severe acute respiratory syndrome coronavirus clade 2 (SARS-CoV-2) has evolved as a significant challenge to the public health of global populations (1). Although various vaccines and antiviral agents are now being developed to reduce virus infection and combat this epidemic, little is known about how viruses interact with their hosts (2, 3). Recent studies have demonstrated a clear genetic link between SARS-CoV-2 infection and COVID-19 severity, and have identified multiple human genomic regions that are linked to disease severity (4, 5). Moreover, COVID-19 patients displayed obvious variations in the immune system, including immune cells, immune checkpoint, and cytokines (6–8). A deeper understanding of the pathogenesis of COVID-19 will facilitate better management of it, and determination of susceptible populations benefit for rationalizing the allocation of medical resources. It is critical and urgent to identify the association between patients' genomes and immune function during viral infections. Accordingly, early detection and appropriate intervention of high-risk patients from a genomic perspective will provide a significant benefit to managing the prevalence of COVID-19.

The N6-methyladenosine (m6A), an innate modification of mRNA and lncRNA, is a reversible procedure regulated by “writers,” “readers,” and “erasers” (9). For its biological characteristics, m6A can regulate carcinogenesis, immunity, stemness, and so on (10–12). Numerous reports have demonstrated that m6A modification serves a prominent part in tumorigenesis through modulating the activity of tumor-associated genes (13, 14). Similarly, m6A is observed and widely studied in diverse virus infections (15, 16), and existing studies have proven the significant role of m6A in the occurrence and progression of COVID-19 (17, 18). However, these researches concentrated predominantly on several m6A-related genes, and a majority of these models were constructed based on non-virally infected cells, which may not fully reveal the authentic status of m6A methylome modifications in immune cells of COVID-19 patients. Therefore, the function of m6A regulators in COVID-19 remain to be further investigated.

In this research, we systematically explored the roles of m6A regulators in the management and categorization of COVID-19. We constructed a gene signature to predict the occurrence of COVID-19 based on 8 selected m6A regulators and observed that patients could benefit from clinical decisions from this signature. Additionally, we identified two m6A subtypes that were closely associated with T-cell activation, indicating that m6A subtypes may distinguish COVID-19 and non-COVID-19 and provide reliable options for clinical treatment.

## Materials and Methods

### Data Collection and Processing

The GSE157103 dataset, composed of 100 COVID-19 patients and 26 non-COVID-19 patients, was acquired from the GEO database (19). This dataset was selected based on some characteristics: sample size >100, diverse disease status, and publicly available data. And all samples are extracted from plasma and leukocyte samples of hospitalized patients. Normalization of the read count values was completed with the limma package (20). A total of 26 m6A regulators was collected from previous studies, and these regulators contain 9 writers, 15 readers, and 2 erasers (Table 1). Differently expressed analysis of these regulators based on limma package was performed between patients with or without COVID-19 to subsequent exploration. A protein-protein interaction (PPI) analysis of differentially expressed genes (DEGs) was performed through the string website (https://cn.string-db.org), and we exhibited gene set variation analysis (GSVA) with the “GSVA” package (21), thus matching the biological function between patients with or without SARS-COV-2 infection.

**Table 1:** m6A modification regulators and their major biological functions.

**Type**	**m6A regulator**	**Function**
Writer	METTL3	Catalyze m6A modification
	METTL14	Facilitate METTL3 recognition of subunits
	METTL16	Catalyze m6A modification
	WTAP	Facilitate METTL3-METTL14 heterodimer to the nuclear speckle
	VIRMA	Bind the m6A complex and mobilize it to specific site
	RBM15	Bind the m6A complex and mobilize it to specific site
	RBM15B	Bind target RNAs and recruiting the WMM complex
	CBLL1	Regulate mRNA splicing and RNA processing
	ZC3H13	Bridge WTAP to the mRNA-binding factor Nito
Reader	YTHDC1	Promote RNA splicing and translocation
	YTHDC2	Promote target RNA translocation
	YTHDF1	Promote RNA translocation
	YTHDF2	Decrease mRNA stability
	YTHDF3	Regulate the translation or degradation
	HNRNPC	Regulate mRNA splicing
	FMR1	Regulate mRNA splicing, stability, dendritic transport and postsynaptic local protein synthesis
	LRPPRC	Regulate nuclear mRNA exportation
	HNRNPA2B1	Promote primary microRNA processing
	IGFBP1/2/3	Recruiting RNA stabilizer
	IGF2BP1	Improve mRNA stability
	ELAVL1	Improve mRNA stability
	RBMX	Regulate gene transcription and pre-mRNAs splicing
Eraser	ALKBH	Regulate mRNA intranuclear transport
	FTO	Catalyze the demethylation of m6A

### Establishment of a Random Forest Model and Support Vector Machine Model

Random forest (RF) and support vector machine (SVM) model was established to predict the prevalence of COVID-19 patients. Several methods, including “Reverse cumulative distribution of residual,” “Boxplots of residual” and receiver operating characteristic (ROC) curve was conducted to validate these models. “RandomForest” package was applied to construct an RF model to identify optimal m6A regulators within the 26 m6A regulators for predicting the prevalence of COVID-19 (22). In this study, to identify optimal RF model, mtry and ntrees were given as 3 and 500 after multiple adjustment. We also discussed the relevance of the 26 m6A regulators and determined the candidate m6A regulators based on 10-fold cross-validation. The Y-axis of the 10-fold cross-validation curve represents the precision of the model when identifying different numbers of m6A regulators. The genes with an importance value over 2 were considered as the disease specific genes for the further analysis. SVM can minimize structural risk, thus enabling classification and regression analysis (23). In SVM model, the expression level of m6A regulators was regarded as the continuous predictive parameter and the sample type was regarded as the categorical variable. The “caret” package was applied to conduct a grid search for the determination of the reasonable hyperparameters for the SVM model with a 5-fold cross-validation (24). Each data is considered as a point in the n-dimensional space (n is 26 in this study), and an appropriate plane was found to distinguish well between the two categories (COVID-19 and non-COVID-19). A repeated 10-fold cross-validation was utilized to tune and evaluate the models. The sample was split into 70% training and 30% test sets. We randomly split the training-test dataset 500 times and used 10-fold repeated 10 times cross-validation approach to optimize the model factors of each round of evaluation. The robustness of these model was assessed based on the area under curve (AUC) value of the receiver operating characteristics (ROC) curve.

### Establishment of the Nomogram

Based on the abovementioned m6A regulators, a nomogram was developed to predict the occurrence of COVID-19 (25). Then, the reliability of this nomogram was assessed by the calibration curve, and decision curve analysis (DCA) was also constructed (26). Moreover, a clinical impact curve was established to evaluate the rationality and benefit of decisions from this nomogram (25).

### Identification of Molecular Subtypes From m6A Regulators

Consensus clustering with K-means algorithms was applied to identify m6A regulators-related subtypes correlated with gene expression (27). The quantity and robustness of clusters were determined with a consensus clustering algorithm realized in the “ConsensuClusterPlus” package (28).

### Identification and Functional Enrichment Analysis of Differentially Expressed Genes

The “limma” package was applied to identify DEGs between different m6A subtypes with the criterion of *p* < 0.001 (29). GO enrichment analysis was utilized to investigate the potential function of the DEGs responsible for COVID-19 with the “clusterProfiler” package (30).

### Establishment of the m6A Gene Signature

Principal component analysis (PCA) was conducted to obtain the m6A score for individual specimens, thus quantifying the m6A subtypes (31). We exhibited the PCA method to identify the m6A subgroups, and the m6A score was acquired based on the following method: m6A score = PC1i, of which PC1 refers to principal component 1 and i to DEG expression (32).

### Exploration of Infiltrating Immune Cell

Single sample gene set enrichment analysis (ssGSEA) was applied to assess the infiltration of immune cells in COVID-19 specimens (33). The gene expression levels in the specimens were sequenced with ssGSEA to acquire an individual grade. We then summarized the expression data of these genes for immunological analysis. Consequently, we gained the enrichment of immune cells in the individual specimen.

### Statistics Analysis

Linear regression analyses were applied to determine the relationship between m6A regulators. Kruskal-Wallis tests were utilized to identify a discrepancy between clusters. All statistical analyses were carried out with two-tailed tests, and the significant value was considered *p* < 0.05. The R software was utilized to perform relevant analysis.

## Results

### Landscape of the 26 m6A Regulators in COVID-19

**Figure 1:**
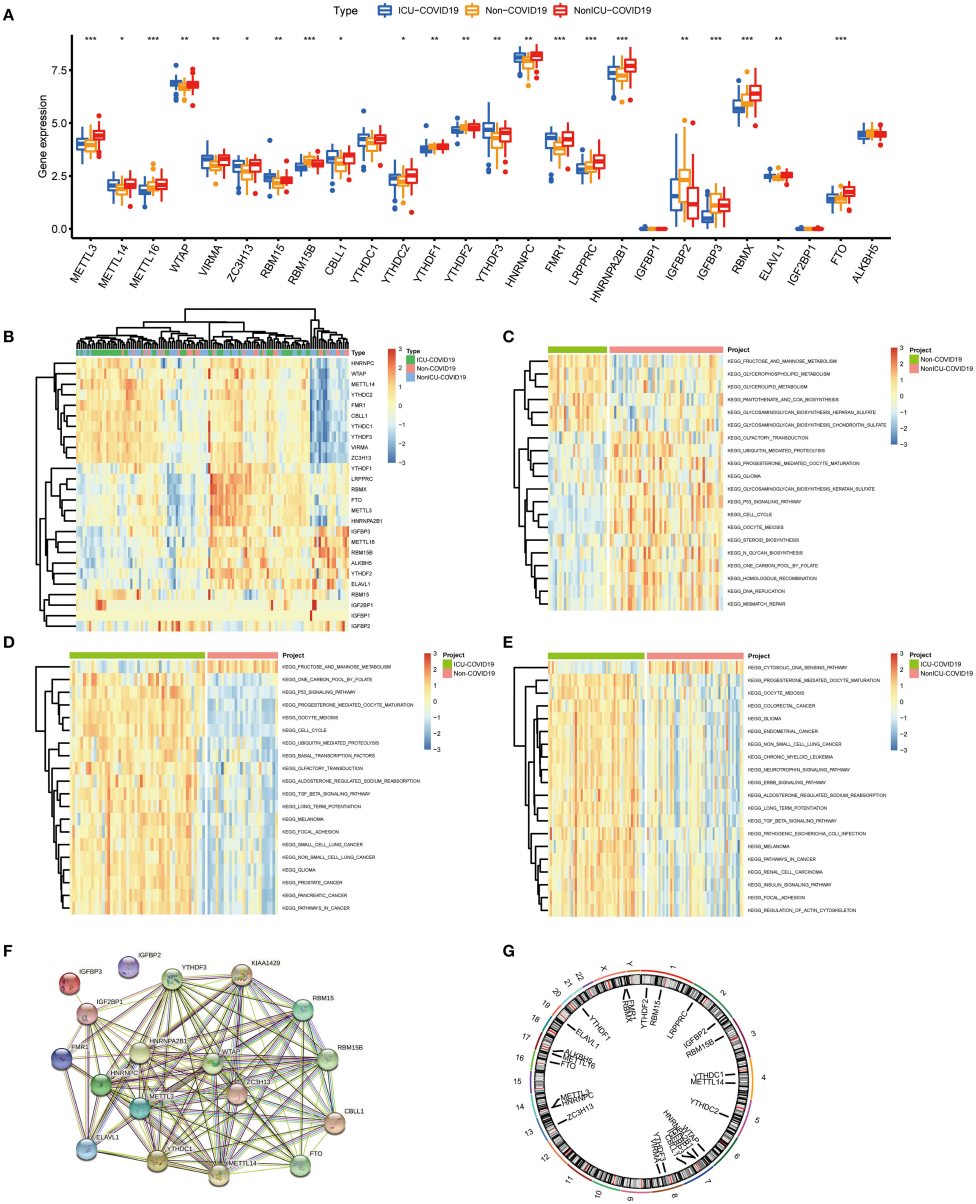
Landscape of the 26 m6A regulators in COVID-19. **(A)** Differential expression analysis of the 26 m6A regulators identified between samples with different COVID-19 status. **(B)** Expression heat map of the 26 m6A regulators in samples. **(C)** GSVA enrichment analysis between Non-COVID-19 and Non-ICU-COVID-19 samples. **(D)** GSVA enrichment analysis between Non-COVID-19 and ICU-COVID-19 samples. **(E)** GSVA enrichment analysis between Non-ICU-COVID-19 and ICU-COVID-19 samples. **(F)** The PPI network analysis among the differentially expressed genes. **(G)** Chromosomal positions of the 26 m6A regulators. *p < 0.05, **p < 0.01, and ***p < 0.001.

Based on the GSE157103 dataset, all samples were divided into three groups (Non-COVID-19, ICU-COVID-19, and Non-ICU-COVID-19). We identified the expression levels of 26 m6A regulators in these groups, of which 22 regulators were differently expressed in these samples. The expression landscape and heatmap of these differentially expressed genes (DEGs) were presented in Figures 1A,B. According to differently expressed analysis of m6A regulators between COVID-19 samples and Non-COVID-19 samples, 18 DEGs were subsequently observed. Most of DEGs were overexpressed in COVID-19 patients compared to non-COVID-19 patients, including METTL3, METTL14, WTAP, VIRMA, ZC3H13, RBM15, CBLL1, YTHDC1, YTHDF3, HNRNPC, HNRNPA2B1, FMR1, ELAVL1, and FTO, and several DEGs, such as RBM15B, IGFBP2, and IGFBP3 were downregulated in COVID-19 patients. Some of DEGs may be associated with the varying severity of COVID-19, such as METTL3, FTO, and RBM15. The finding was consistent with previous reports (17, 34, 35). We further conducted GSVA analysis to explore the biological difference between different groups. Compared to samples without COVID-19, p53 signaling pathway, cell cycle, oocyte meiosis, and olfactory transduction were obviously enriched in COVID-19 samples (Figures 1C,D). Similarly, we observed that diverse signaling pathways were more enriched in the ICU-COVID-19 samples than Non-ICU-COVID-19 samples, such as oocyte meiosis, ERBB signaling pathway, and TGF-β signaling pathway (Figure 1E). These results demonstrated that identified signaling pathways were potentially associated with the occurrence and severity of COVID-19. A protein-protein interaction (PPI) analysis was also performed to show the interactivity of DEGs, which demonstrated that METTL3 and YTHDF3 were hub genes (Figure 1F). Additionally, the location of m6A regulators on the chromosome was discussed and displayed in Figure 1G.

### Association Between Writers and Erasers in COVID-19

**Figure 2:**
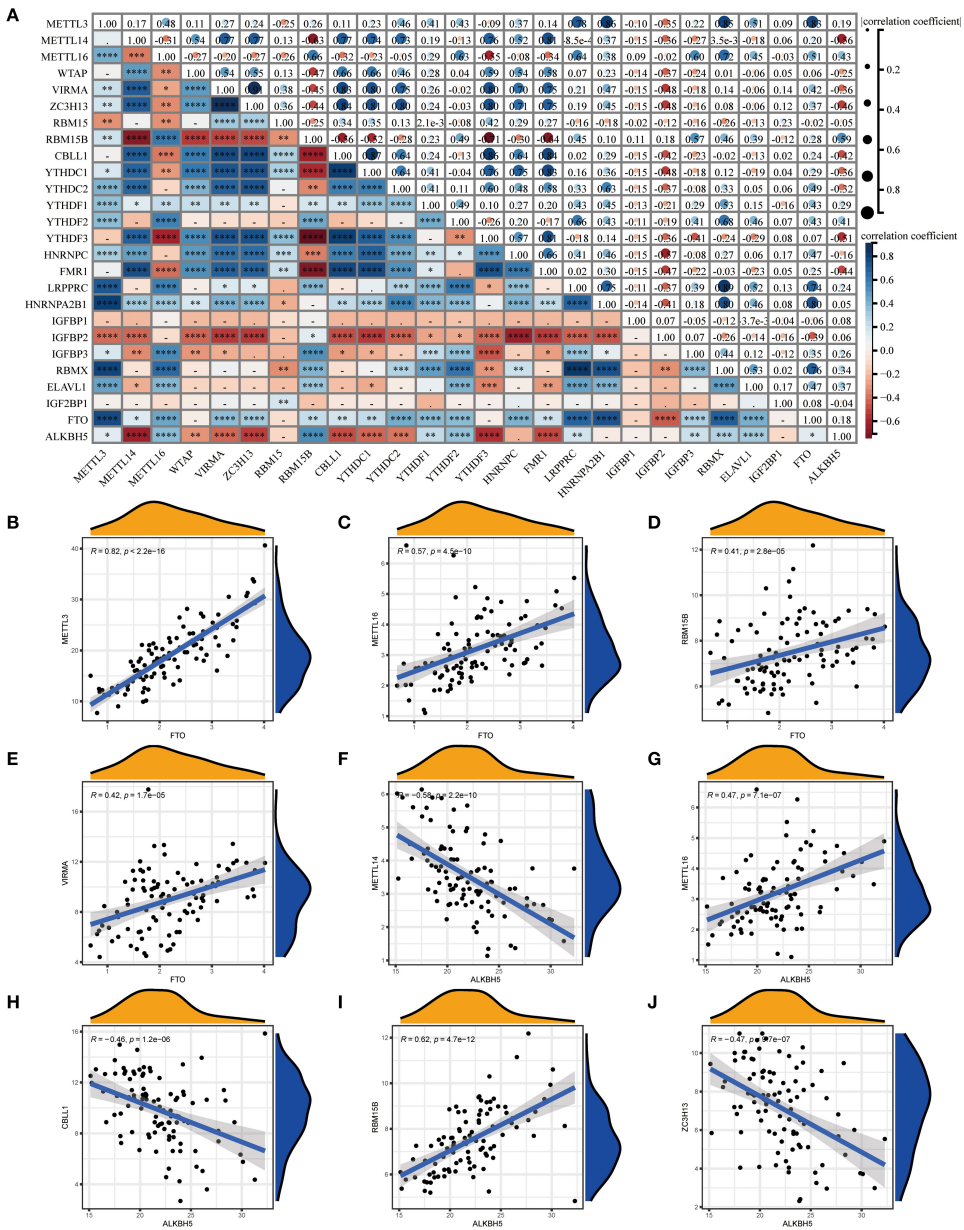
Correlation between m6A regulators in COVID-19. **(A)** Correlation plot of 26 m6A regulators. **(B–J)** Correlation between writers and erasers in COVID-19. Writer genes: METTL3, METTL14, METTL16, RBM15B, VIRMA, CBLL1, and ZC3H1; eraser genes: ALKBH5 and FTO. *p < 0.05, **p < 0.01, and ***p < 0.001.

We investigated the correlation between three types of m6A modification, and the result was presented in Figure 2A. Interestingly, m6A regulators of a different type, such as METTL3 and HNRNPA2B1, can display cooperative activities (coefficient = 0.86). We also discussed the possibility of regulators co-expression, and observed a clear relationship between FTO and additional regulators, with the greatest relevance for METTL3 and FTO (correlation coefficient = 0.83). This finding is consistent with PPI analysis and provides a possible explanation for the regulation mechanism of m6A regulators. To further investigate the relationship between writers and erasers in COVID-19, we discussed the expression levels of these regulators with linear regression analyses. Significant positive correlations were observed between METTL3, METTL16, RBM15B, VIRMA, and FTO in COVID-19 patients. COVID-19 patients with high expression levels of FTO tend to display high levels of METTL3, METTL16, RBM15B, or VIRMA (Figures 2B–E). Similarly, we also found a close association between CBLL1, METTL14, METLL16, RBM15B, ZC3H13, and ALKBH5. COVID-19 patients with elevated expression levels of CBLL1, METLL16, and RBM15B presented elevated expression levels of ALKBH5 while elevated METTL14 and ZC3H13 expression demonstrated a negative association with ALKBH5 (Figures 2F–J). Consequently, we proved a clear association between diverse writers and erasers.

### Evaluation of the RF Model and SVM Model

**Figure 3:**
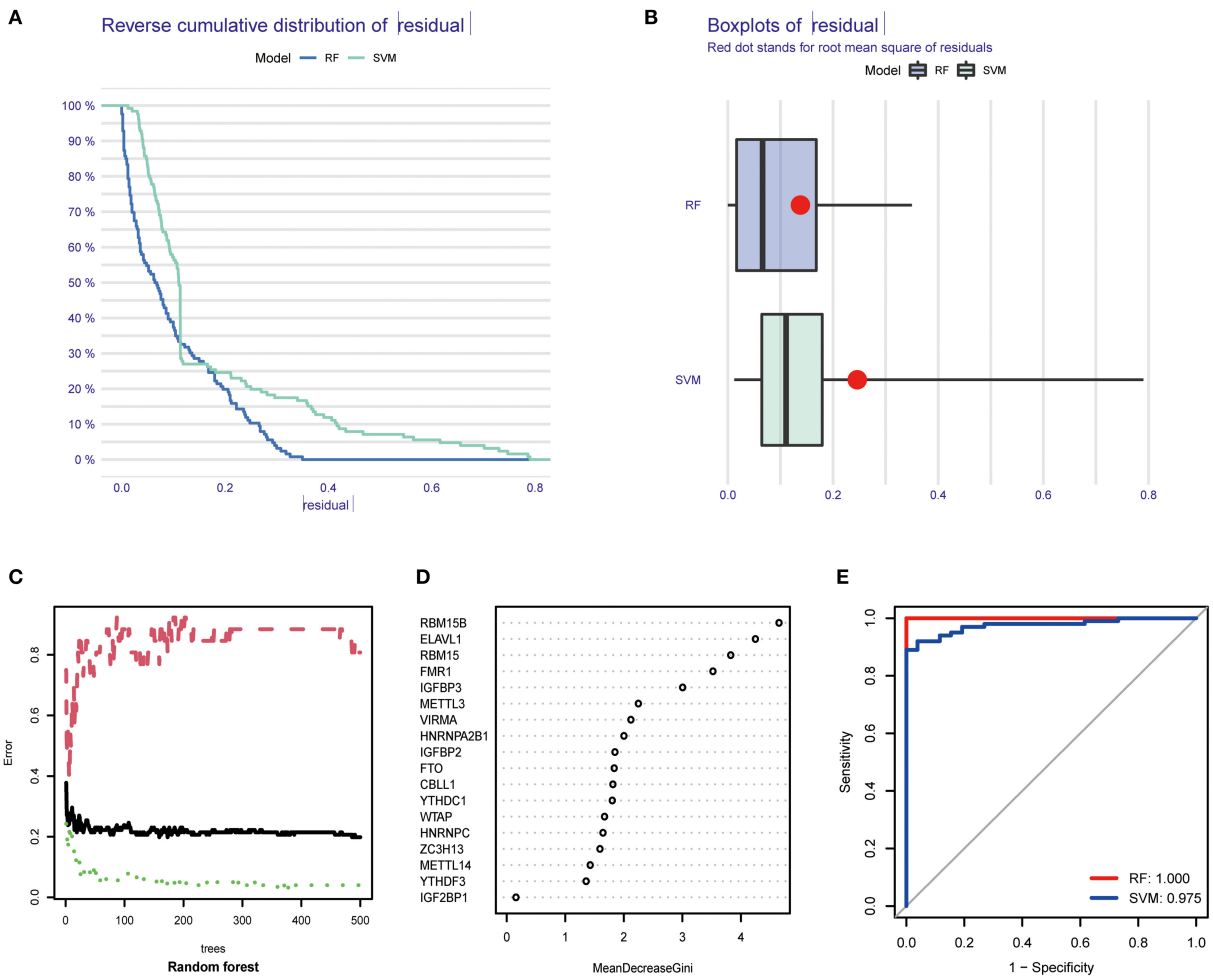
Establishment of RF model and SVM model. **(A)** Reverse cumulative distribution of residual was displayed to demonstrate the residual distribution of RF and SVM model. **(B)** Boxplots of residual was displayed to demonstrate the residual distribution of RF and SVM model. **(C)** The influence of the number of decision trees on the error rate. **(D)** The importance of the 26 m6A regulators based on the RF model. **(E)** ROC curves revealed the accuracy of the RF and SVM model.

We next constructed an RF and SVM model to identify optimal m6A regulators from abovementioned DEGs to predict the occurrence of COVID-19. Based on “Reverse cumulative distribution of residual” and “Boxplots of residual” (Figures 3A,B), the RF model with the least residuals were established. As a majority of the specimens in this model retained only small residuals, the predictive performance of the RF model is extremely excellent. Then, we chose 500 trees as the variables of the current model based on the relationship overview between the model error and the number of decision trees, and this model presented a stable error possibility (Figure 3C). We also ranked 18 DEGs depending on their respective gene importance based on RF model, and this result demonstrated that RBM15B and ELAVL1 had a high priority in this model (Figure 3D). Additionally, the ROC curves were established to assess the accuracy of these models, and the AUC value also demonstrated that the RF model has superior performance compared to the SVM model (Figure 3E).

### Evaluation of a Predictive Nomogram

**Figure 4:**
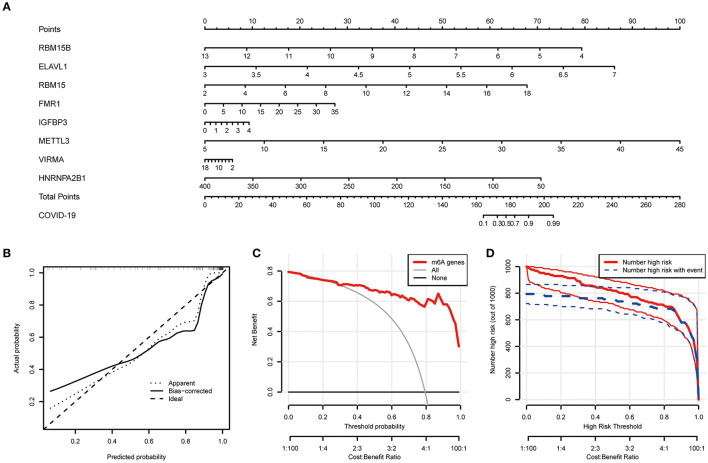
Establishment of the nomogram model. **(A)** Establishment of the nomogram model based on the 8 selected m6A regulators. **(B)** Predictive robustness of the nomogram model as disclosed by the calibration curve. **(C)** Decisions based on the nomogram model may benefit COVID-19 patients. **(D)** Clinical impact of the nomogram model as evaluated by the clinical impact curve.

Based on the abovementioned findings, 8 recommended m6A regulators were utilized to develop a predictive nomogram for predicting the incidence of COVID-19 (Figure 4A). Interestingly, we observed that the expression level of RBM15B was negatively correlated with the patients' risk score, and RBM15B may be a protective factor for COVID-19 patients. This result was consistent with abovementioned analysis based on the expression difference in the patients with different disease status. Calibration curves proved the predictive accuracy of the nomogram (Figure 4B). The model developed by the m6A regulator is always at the top of the DCA curve (Figure 4C), indicating that COVID-19 patients were clearly benefited from the decisions based on this nomogram. Furthermore, the clinical impact curve also demonstrated that the predictive robustness of this nomogram was reliable (Figure 4D).

### Analysis of Specific Subtypes Based on m6A Regulators

**Figure 5:**
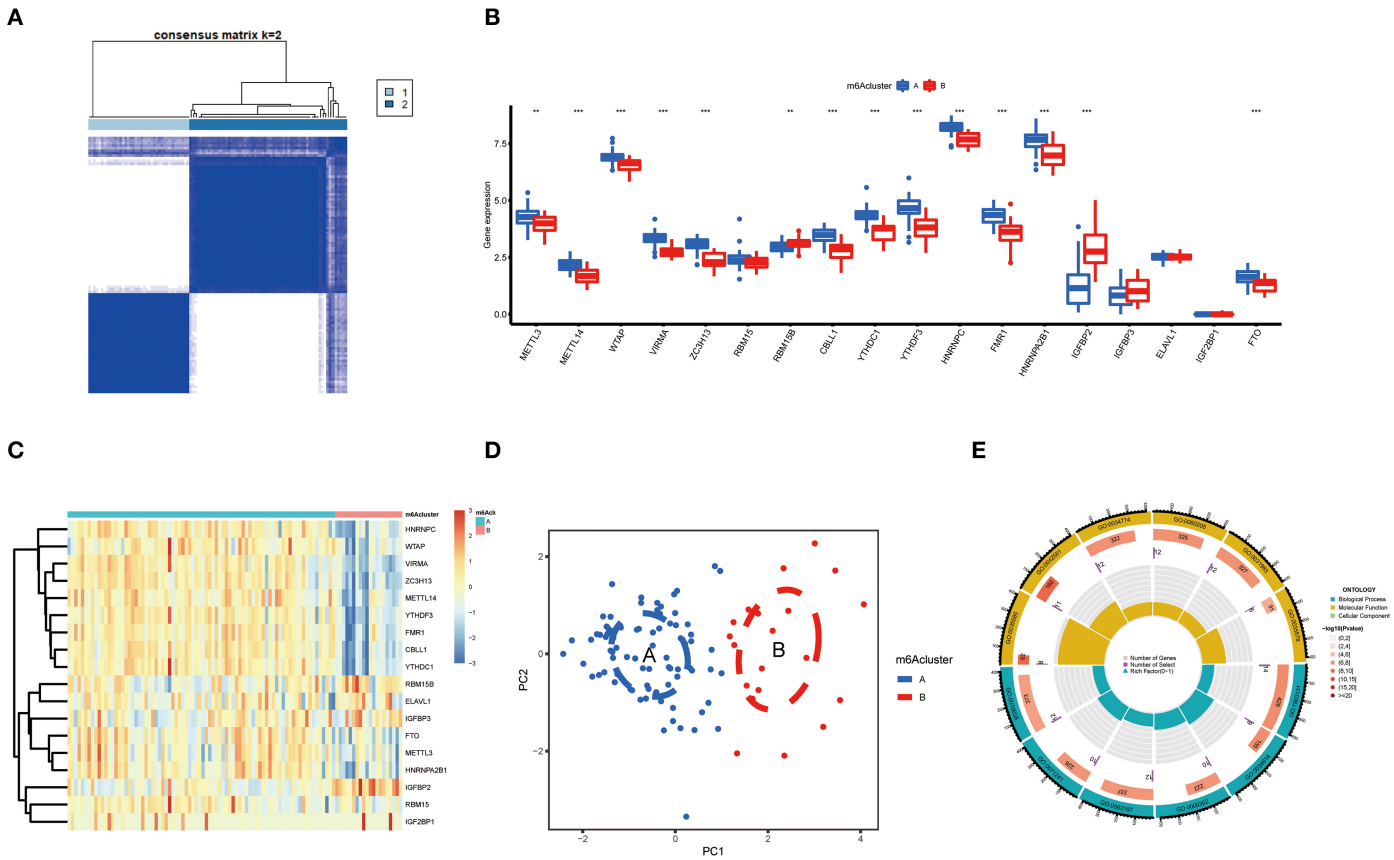
Consensus clustering of the 18 significant m6A regulators in COVID-19. **(A)** Consensus matrices of the 18 significant m6A regulators for *k* = 2. **(B)** Differential expression analysis of the 18 significant m6A regulators in cluster A and cluster B. **(C)** Expression heatmap of the 18 significant m6A regulators in cluster A and cluster B. **(D)** PCA for the expression data of the 18 significant m6A regulators that indicates an obvious difference in transcriptomes between the two m6A subtypes. **(E)** GO analysis that investigates the potential mechanism underlying the effect of the 139 m6A-related DEGs on the occurrence and development of COVID-19. *p < 0.05, **p < 0.01, and ***p < 0.001.

Based on differently expressed m6A regulators, we performed a consensus clustering algorithm to identify different subtypes (Figure 5A), and COVID-19 patients were well-categorized into two clusters when the cluster variable is 2. Cluster A consisted of 80 cases, and cluster B consisted of 20 cases. Subsequently, we detected the expression of these m6A regulators in cluster A and Cluster B. METTL3, METTL14, WTAP, VIRMA, ZC3H13, CBLL1, YTHDC1, YTHDF3, HNRNPC, FMR1, HNRNPA2B1, and FTO presented increased expression in cluster A compared to those in the cluster B, while the opposite performance was observed in IGFBP2. Meanwhile, RBM15, RBM15B, IGFBP3, ELAVL1, and IGF2BP1 displayed no significant differences between these clusters (Figures 5B,C). PCA revealed that the 18 m6A regulators could exactly classify the two m6A subtypes (Figure 5D). Totally, 139 m6A-related DEGs were identified between the two m6A subtypes. To explore the potential role of these DEGs in COVID-19, the findings from GO enrichment analysis revealed that the DEGs were particularly abundant in cellular response and cell differentiation-related pathways (Figure 5E).

**Figure 6:**
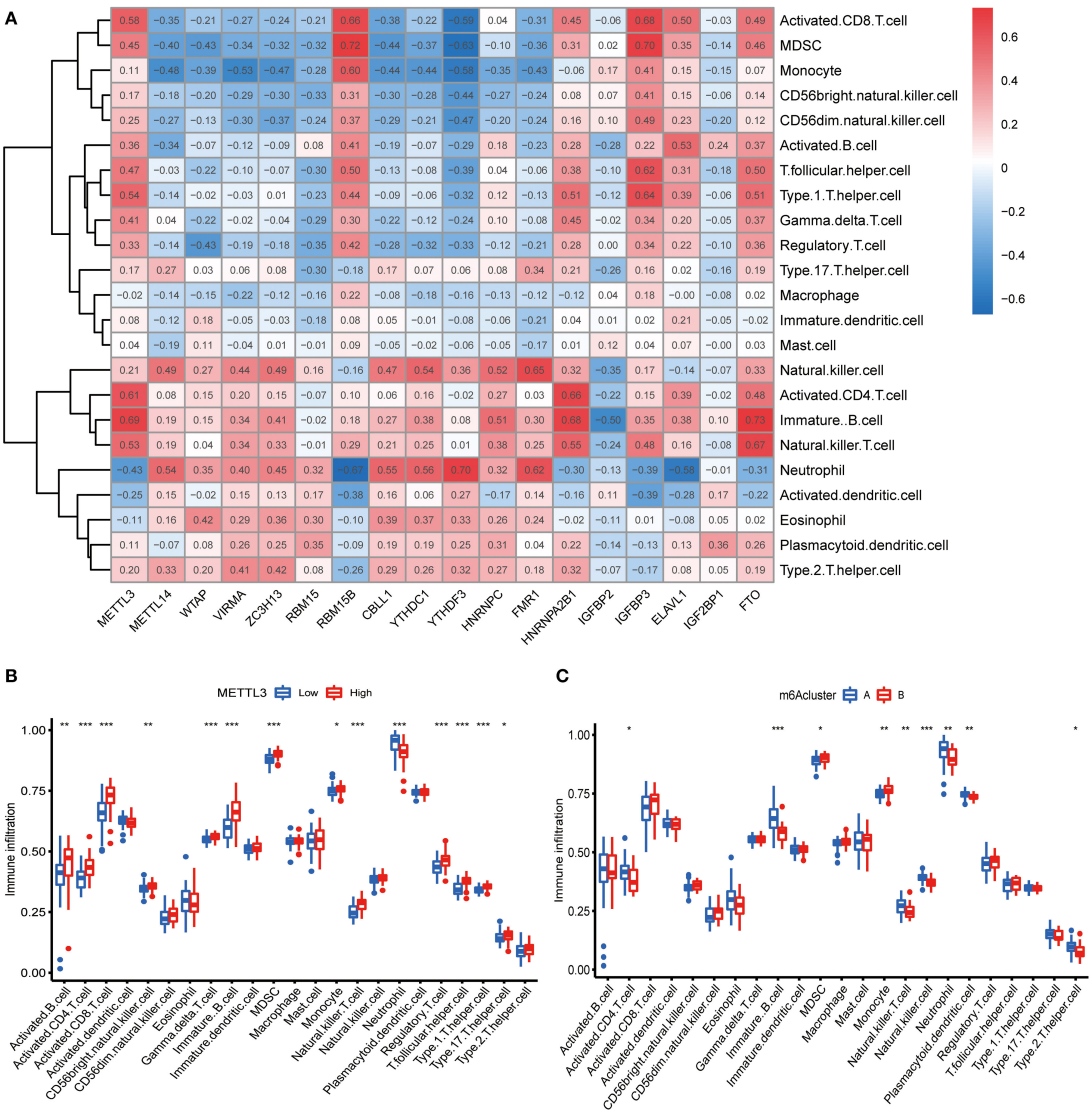
Single sample gene set enrichment analysis. **(A)** Correlation between infiltrating immune cells and the 18 significant m6A regulators. **(B)** Difference in the abundance of infiltrating immune cells between high and low METTL3 expression groups. **(C)** Differential immune cell infiltration between cluster A and cluster B. *p < 0.05, **p < 0.01, and ***p < 0.001.

We further conducted ssGSEA to assess the enrichment of immune cells in COVID-19 specimens and discussed the relationship between the m6A regulators and immune cells (Figure 6A). METTL3 had positive associations with various immune cells. Afterward, we investigated the distinct enrichment of immune cells in patients with high- or low-METTL3 (Figure 6B). The findings demonstrated that patients with high METTL3 expression had obviously enriched immune cells. Ultimately, we also discussed the differential immune cell enrichment between the m6A subtypes. We observed that cluster A displayed higher infiltrating levels of immune cells, particularly T helper cells (Th1 and Th2), than cluster B (Figure 6C), which indicated that patients in cluster A may have a positive immune response for COVID-19.

### Evaluation of the m6A Gene Signature

**Figure 7:**
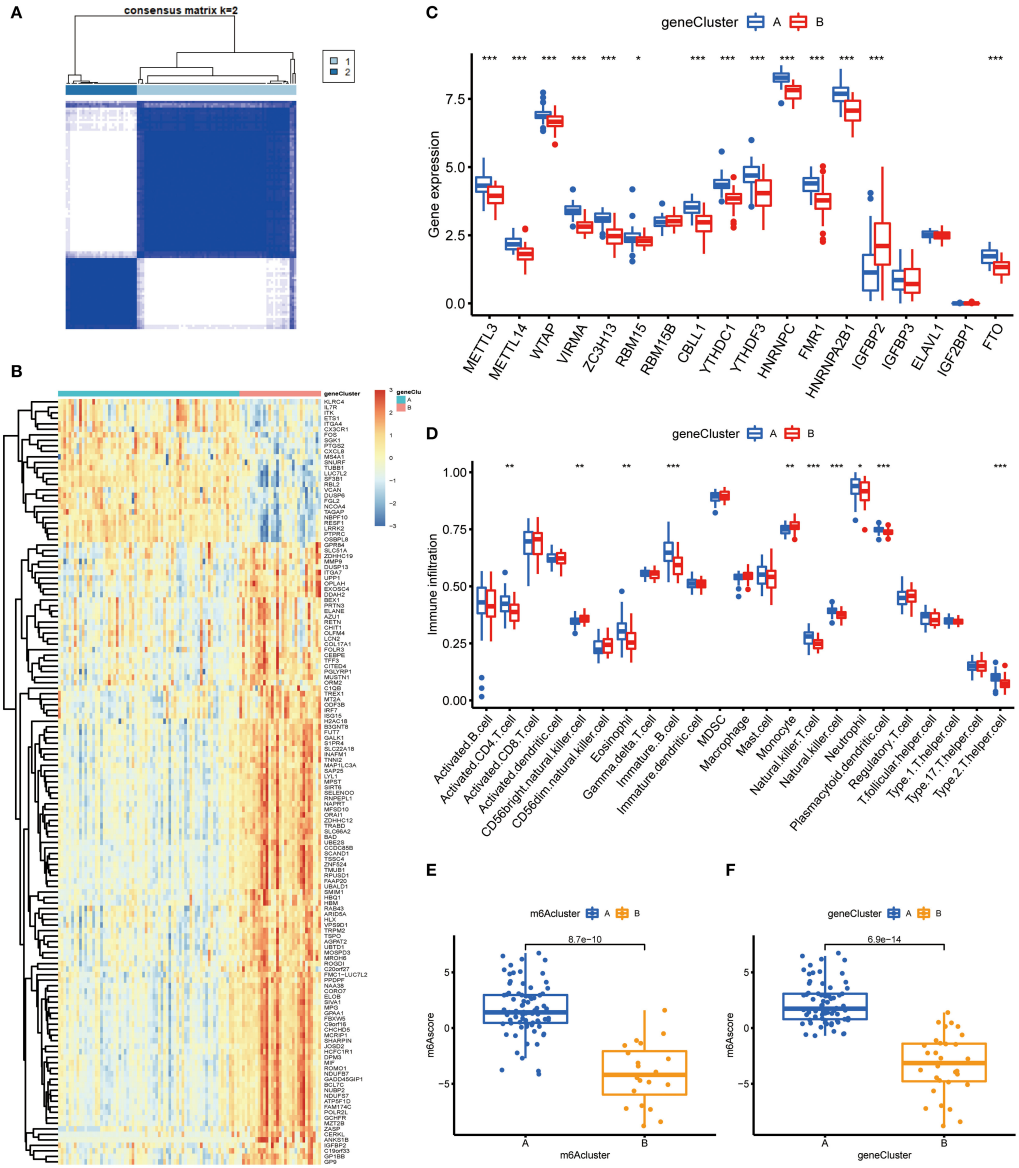
Consensus clustering of the 139 m6A-related DEGs in COVID-19. **(A)** Consensus matrices of the 139 m6A-related DEGs for *k* = 2. **(B)** Expression heat map of the 139 m6A-related DEGs in gene cluster A and gene cluster B. **(C)** Differential expression of the 18 significant m6A regulators in gene cluster A and gene cluster B. (D) Differential immune cell infiltration between gene cluster A and gene cluster B. **(E)** Differences in m6A score between cluster A and cluster B. **(F)** Differences in m6A score between gene cluster A and gene cluster B. *p < 0.05, **p < 0.01, and ***p < 0.001.

**Figure 8:**
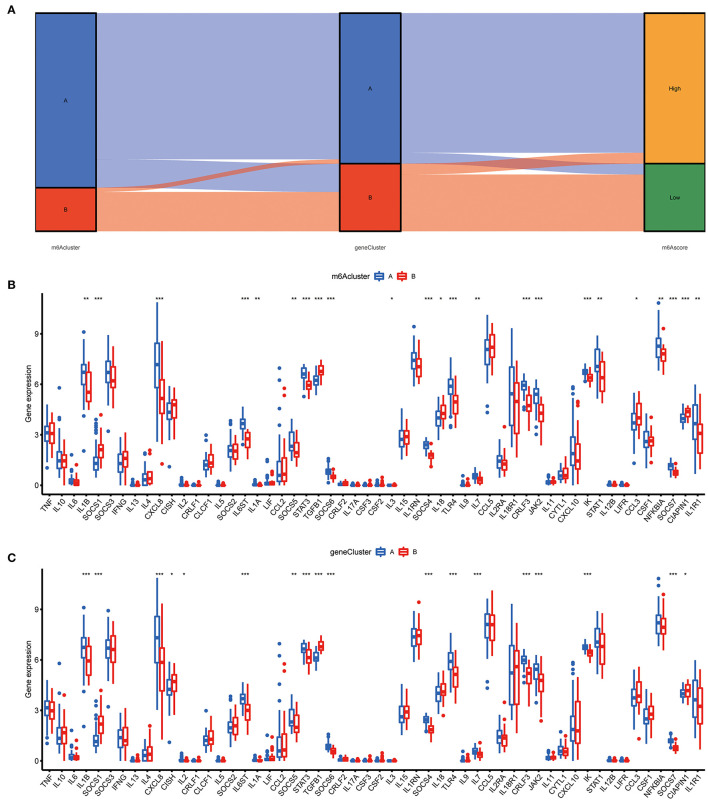
Role of m6A subtypes in distinguishing COVID-19. **(A)** Sankey diagram demonstrating the relationship between m6A subtypes, m6A gene subtypes, and m6A scores. **(B)** Differential expression levels of cytokines between cluster A and cluster B. **(C)** Differential expression levels of cytokines between gene cluster A and gene cluster B. *p < 0.05, **p < 0.01, and ***p < 0.001.

To prove the m6A subtypes, we performed the consensus clustering algorithm to categorize the COVID-19 patients into distinct gene subgroups based on 139 m6A-related DEGs (Figure 7A). We observed that these genomic subtypes were in accordance with m6A subtypes, and Figure 7B displayed the differential expression of the 139 DEG. Afterward, the differential expression of the 18 m6A regulators and infiltrating immune cells between different gene clusters were also similar to those in the m6A subtypes (Figures 7C,D). This result demonstrated the rationality of the clustering algorithm. Moreover, PCA was utilized to obtain m6A scores for individual specimens, thus quantifying the m6A subtype. We also compared the m6A score in the m6A clusters or gene clusters, and the finding revealed the m6A score in cluster A or gene cluster A was greater than that in cluster B or gene cluster B (Figures 7E,F). Additionally, the correlation between the m6A cluster, m6A gene clusters, and m6A scores were displayed in Figure 8A.

### Relationship Between m6A Subtypes and Cytokines

The “cytokine storm” is an inappropriate immune response that is the main cause of death in COVID-19, and many cytokines and their inhibitors are now used in the clinical treatment of COVID-19. To further determine the correlation between m6A subtypes and COVID-19, we comprehensively discussed the association between m6A subtypes and various cytokines. As displayed in Figures 8B,C, diverse cytokines presented significant discrepancies in the m6A clusters and genomic clusters. It is noteworthy that IL1B, IL7, IL8, and IL6ST were overexpressed in the cluster A and gene cluster A compared to cluster B and gene cluster B, consistent with existing reports. This finding revealed that cluster A or gene cluster A is closely correlated with COVID-19 characterized by multiple cytokines.

## Discussion

COVID-19 is an infectious respiratory disease with general susceptibility in the population, and there are limited treatment strategies for COVID-19 at present (36). To improve the management and recovery of patients with limited medical facilities, it is essential to clarify the pathogenesis of COVID-19 and the associated susceptible population. Emerging evidence demonstrated that m6A regulators participate in the diverse biological behavior of SARS-COV-2 (18, 37). However, the potential role of m6A regulators in the COVID-19 is still unclear.

In the present research, we comprehensively explored the basic elements of m6A modification in COVID-19 patients. The expression levels of m6A regulators were obviously overexpressed in COVID-19 patients compared to in non-COVID-19 patients. This different expression of m6A regulators was also observed between COVID-19 patients with ICU status and non-ICU status. These results indicated that m6A modification may have a close correlation with development and severity of COVID-19. We also performed GSVA to identify COVID-19-related pathways and found diverse signaling pathways may serve a critical role in the development of COVID-19, and the exploration of these pathways may be beneficial for clarifying the special mechanism of COVID-19. We further discussed the intrinsic relevance of m6A regulators in the patients with or without COVID-19, and a significant association between m6A regulators in COVID-19 was observed. Moreover, an RF model was constructed to identify 8 regulators from differential expressed m6A regulators and thus predict the occurrence of COVID-19. However, this model cannot yet be validated in the absence of adequate information of m6A regulators in the public databases. Additionally, univariate analysis for feature selection had a possibility to ignore the multivariate association in the feature selection process, and multivariate analysis was further considered to identify optimal DEGs. Previous reports have demonstrated that the selected m6A regulators are responsible for the initiation and progression of tumors, such as hepatocellular carcinoma, lung cancer, and gastric cancer (32, 38, 39). Currently, there are few studies on the correlation between these selected regulators and COVID-19. This study provides a novel option for further genomic analysis on these m6A regulators in the COVID-19 patients.

A multicomponent m6A methyltransferase complex (MTC) consisted of a METTL3-METTL14 heterodimer core and additional binding elements (40). MTC can promote m6A modification to regulate the disease processes. A nomogram based on 8 candidate m6A regulators was constructed to guide clinical treatment for COVID-19 patients, and the DCA curve demonstrated that COVID-19 patients may benefit from the decisions based on this nomogram. We observed that RBM15B, HNRNPA2B1, and VIRMA may be protective factors in the development of COVID-19, and the opposite performance was found in ELAVL1, RBM15, FMR1, IGFBP3, and METTL3. RBM15 and its paralogue RBM15B bind the m6A-methylation compound and mobilize it to appropriate sites in RNA (41). RBM15 was markedly upregulated in laryngeal squamous cell carcinoma and correlated with a worse prognosis (42). METTL3 serves a critical role in various cellular biological processes, such as promoting the anti-tumor immunity of natural killer cells (43). As a prominent subunit of the MTC, METTL3 facilitates the generation of m6A. It is reported that METTL3 and RBM15 can modulate intrinsic immune responses of the host cell during SARS-CoV-2 infection in diverse cells (18). Similarly, the specific role of VIRMA, ELAVL1, and FMR1 in COVID-19 was mentioned in several studies (44–46). Numerous studies demonstrated that the 8 selected m6A regulators may be involved in the emergence and lymphocyte responses of COVID-19 patients.

At present, the immune response activated by T cells may benefit COVID-19 patients, and reduce the damage caused by cytokine storms (47, 48). Based on DEGs between COVID-19 and non-COVID-19, we found 18 m6A regulators for subsequent analysis. Unsupervised cluster analysis of differential expressed m6A regulators was performed to identify two distinct modification subtypes in COVID-19 patients. m6A cluster A presented activated T cell behaviors, while m6A cluster B was marked by monocyte-related activity. Similar to the m6A categorization, two genomic subtypes were established based on DEGs between cluster A and cluster B, and we found that gene cluster A displayed higher infiltrating levels of T cells than gene cluster B, such as CD4+ T cells and natural killer T cells. JAK-STAT pathway may participate in T cell differentiation (49), and we observed that components in the JAK-STAT pathway were more enriched in cluster A or gene cluster A than those in cluster B or gene cluster B. Consequently, these findings demonstrated that m6A cluster A and gene cluster A with positive T cell activity to defend against SARS-COV-2 could present a superior clinical performance. Furthermore, the m6A score was identified to quantify the m6A subtype for individual COVID-19 patients. Consistent with the above results, patients in m6A cluster A or gene cluster A displayed higher m6A scores compared to m6A cluster B or gene cluster B.

Nonetheless, there are some limitations in the present research. Since our findings have not been supported by clinical specimens, the specific relationship between m6A regulator and COVID-19 remains to be further confirmed. And this signature will be evaluated and validated in future experimental studies.

## Conclusion

Briefly, this research identified 8 recommended m6A regulators and constructed a nomogram that predicts the susceptibility of COVID-19. Based on differently expressed m6A regulators, we then determined two m6A subtypes, and cluster B may be clearly associated with COVID-19.

## Data Availability Statement

The original contributions presented in the study are included in the article/supplementary material, further inquiries can be directed to the corresponding author.

## Author Contributions

XQ performed data collection and analysis. XQ and QC wrote the manuscript. KW polished and revised the manuscript. All authors contributed to the study's conception and design. All authors commented on previous versions of the manuscript, read and approved the final manuscript.

## Conflict of Interest

The authors declare that the research was conducted in the absence of any commercial or financial relationships that could be construed as a potential conflict of interest.

## Publisher's Note

All claims expressed in this article are solely those of the authors and do not necessarily represent those of their affiliated organizations, or those of the publisher, the editors and the reviewers. Any product that may be evaluated in this article, or claim that may be made by its manufacturer, is not guaranteed or endorsed by the publisher.
